# Adaptation and implementation of a clinical protocol for pre-eclampsia with severe features and eclampsia in a teaching hospital in Ghana: a study protocol

**DOI:** 10.62675/2965-2774.20260312

**Published:** 2026-07-21

**Authors:** Moses Siaw-Frimpong, Abigail Beane, Rashan Haniffa, Jorge Ibrain Figueira Salluh, John Humphrey Amuasi, Yaw Gyanteh Owusu, Fathima Fazla, Joseph Bonney, Ibrahim Kwaku Duah, Patience Adjepong, Nana Fosua Gyapon, Irene Bandoh, William Addison, Andrew Panyin Vormawor, Charles Adu-Takyi, Augustine Tawia

**Affiliations:** 1 Komfo Anokye Teaching Hospital Directorate of Anaesthesia and Intensive Care Kumasi Ghana Directorate of Anaesthesia and Intensive Care, Komfo Anokye Teaching Hospital - Kumasi, Ghana.; 2 University of Edinburgh Institute of Regeneration and Repair Pandemic Science Hub Edinburgh United Kingdom Pandemic Science Hub, Institute of Regeneration and Repair, University of Edinburgh - Edinburgh, United Kingdom.; 3 Instituto D’Or de Pesquisa e Ensino Rio de Janeiro RJ Brazil Instituto D’Or de Pesquisa e Ensino - Rio de Janeiro (RJ), Brazil.; 4 Kumasi and Bernhard Nocht Institute of Tropical Medicine Kwame Nkrumah University of Science and Technology Hambur Germany Kwame Nkrumah University of Science and Technology, Kumasi and Bernhard Nocht Institute of Tropical Medicine - Hamburg, Germany.; 5 Komfo Anokye Teaching Hospital Directorate of Obstetrics and Gynaecology Kumasi Ghana Directorate of Obstetrics and Gynaecology, Komfo Anokye Teaching Hospital - Kumasi, Ghana.; 6 Mahidol Oxford Tropical Medicine Research Unit Nat-Intensive Care Surveillanc Colombo Sri Lanka Nat-Intensive Care Surveillance, Mahidol Oxford Tropical Medicine Research Unit - Colombo, Sri Lanka.; 7 Kumasi Centre for Collaborative Research in Tropical Medicine Global Health and Infectious Disease Research Group Kumasi Ghana Global Health and Infectious Disease Research Group, Kumasi Centre for Collaborative Research in Tropical Medicine - Kumasi, Ghana.

**Keywords:** ICU management, Pre-eclampsia with severe features, Eclampsia, Clinical management protocol

## Abstract

**Background:**

Pre-eclampsia is a multisystemic disorder characterized by varied degrees of placental malperfusion. It is estimated to affect 3 - 5% of pregnancies globally, accounting for up to 15% of maternal morbidity and mortality. Solutions to improve outcomes for pre-eclampsia and eclampsia are increasingly focused on improving recognition and on timely, effective treatment, notably through the implementation of treatment guidelines and protocols. To date adoption of guidelines in clinical practice have been variable, with the lowest adoption observed in settings where the policies and evidence did not originate. This study is designed to address known barriers by proposing a stakeholder-led, co-designed protocol adaptation process, followed by an evaluation of the effectiveness of a multi-implementation strategy (education, audit and feedback, and the use of champions) to support practice change.

**Methods:**

Using co-design, a protocol for managing pre-eclampsia with severe features and eclampsia will be adapted and implemented. The process will involve three stages; protocol selection and adaptation, implementation of the clinical protocol and evaluation of the process. The primary outcome will be implementation success, assessed across three domains of the Reach, Effectiveness -Adoption Implementation and Maintenance (RE-AIM) framework: Fidelity, Reach, and Adoption. Secondary outcomes, including intervention effectiveness and clinical safety endpoints, will also be evaluated.

**Analysis:**

The individual components of the RE-AIM framework will be computed as percentages. A composite threshold of 80% will be deemed success. The secondary outcome variables will be compared to the pre-implementation period.

## INTRODUCTION

Pre-eclampsia is a multisystemic disorder characterized by varied degrees of placental malperfusion.^(1)^ It is diagnosed when there is maternal hypertension coupled with new-onset proteinuria presenting on or after 20 weeks of gestation.^(2)^ Pre-eclampsia is estimated to affect 3 - 5% of pregnancies globally^(3)^ accounts for up to 15% of maternal morbidity and mortality.^(4)^ Incidence and associated morbidity and mortality is disproportionately higher in low and lower-middle-income countries affecting up to 151 in every 10,000 deliveries.^(3)^

Preeclampsia is diagnosed by a constellation of signs and symptoms, and presents as a spectrum in severity. In its simplest presentation, it is diagnosed in the presence of maternal hypertension coupled with new-onset proteinuria presenting on or after 20 weeks of gestation.^(2)^ Severe pre-eclampsia and presentation includes; non-invasive blood pressure being ≥ 160/110mmHg measured on two occasions at least 4 hours apart, the presence of thrombocytopenia (platelets less than 100,000/mL), severe persistent right upper quadrant or epigastric pain, abnormally elevated liver enzymes, progressive renal insufficiency, pulmonary oedema, and new-onset visual or cerebral disturbance.^(2)^ At its most severe, eclampsia which is defined as the occurrence of one or more generalized tonic-clonic convulsions unrelated to other medical conditions in women with hypertensive disorder of pregnancy.^(5)^ Pre-eclampsia is estimated to affect 3 - 5% of pregnancies globally.^(3)^ In Ghana, the incidence estimates vary from 2.9%^(5)^ to 9.9%.^(6)^ The incidence and burden on poor outcomes are as a result of both population characteristics and quality of and access to maternal, antenatal and obstetric services in the country.^(4)^

Potential solutions to improve outcomes in the presence of pre-eclampsia and eclampsia increasingly focus on improving recognition and on timely, effective treatment, notably through the implementation and adoption of treatment guidelines and protocols.^(7)^ A review of pregnancy-related deaths arising from pre-eclampsia/eclampsia recommended the need for hospitals and facilities to adopt and implement standard policies and protocols in the management of pre-eclampsia and eclampsia to improve outcomes.^(8)^

Evaluations of existing care quality in relation to maternal management have established the poor uptake of scientific evidence of best practices in clinical care.^(9)^ Effective methods to support implementation and enhance adoption of evidence-based practices in daily care to improve patient outcomes are warranted.^(10)^ Where implementation has been most successful, approaches have included stakeholders’ participation, contextual adaptation to policies, and efforts to increase ownership from clinicians.^(11)^

This protocol describes a proposed study to evaluate a co-designed, stakeholder-led implementation of an evidence-based protocol for the management of pre-eclampsia in patients presenting to the Emergency Department (ED) at a hospital in Ghana. Safety endpoints and clinical outcomes will also be pre-determined and evaluated.

### Objectives

First, this study will assess the feasibility and impact of implementing a clinician-co-designed and context-adapted protocol for managing patients with pre-eclampsia and eclampsia.

Secondly, the study aims to evaluate the potential impact of the protocol on clinical care processes and outcomes (including safety endpoints).

The study will identify barriers and facilitators to implementing a clinician-co-designed protocol for managing pre-eclampsia with severe features and eclampsia, as reported by healthcare professionals (doctors, Nurses/Midwives, and pharmacists), and report adaptations to the protocol design and implementation strategy to overcome these barriers. To achieve these aims, the study combines Medical Research Council (MRC) recommended guidance for stakeholder-led protocol development, with an implementation evaluation tool, the Reach, Effectiveness -Adoption Implementation and Maintenance (RE-AIM) framework.^(12)^

## METHODS

### Ethical consideration

The Institutional Review Board of the Komfo Anokye Teaching Hospital has approved this study as part of a set of quality-improvement interventions to enhance maternal outcomes. The interventions are intended to improve service delivery by using evidence-based recommended practices, and successful implementation is determined by the intervention's reach, i.e., the proportion of eligible patients who receive it; therefore, no individual patient consent will be required.

### Study design

This will be an implementation-effectiveness study with the primary outcomes being determinants of implementation success. These are described below.

### Study site

The study will be conducted at the Obstetric Emergency Unit of the Komfo Anokye Teaching Hospital. The Directorate of Obstetrics and Gynecology of the Komfo Anokye Teaching Hospital in Ghana. Komfo Anokye Teaching Hospital is a 1,500-bed facility in the Northern half of Ghana. The Directorate of Obstetrics and Gynecology is a 250-bed department with a 35-bed Emergency Unit. The annual deliveries at the hospital average 4,500 - 5,000. There is currently no protocol for managing these emergency cases, leading to variations in clinical care.

### Study participants

The study will include all patients who present at the obstetric emergency with the clinical assessment of pre-eclampsia with severe features or eclampsia. Severe features are defined as the presence of one of the following; platelets, 100 x 109, severe persistent right upper quadrant pain, renal insufficiency (serum creatinine > 1.1mg/dL or doubling of serum creatinine in the absence of renal disease), pulmonary oedema, visual disturbance, unexplained new-onset headache unresponsive to medication and HELLP syndrome acronym from hemolysis, elevated liver enzymes and low platelets.^(13)^

Patients who present dead on arrival to the emergency unit will be excluded. There are a few patients who will not present to the ED with pre-eclampsia/eclampsia but may develop the condition days later during a hospital admission for another pregnancy-related condition. These patients will also be excluded, given the variation in their hospital journey and the likely limited number of affected patients.

### Study procedure

The study will employ the principles of co-design in all phases, from the clinical protocol for managing pre-eclampsia with severe features and eclampsia through implementation, adaptations, dissemination, and learning following evaluation. Co-design is a collaborative methodology that actively engages a broad range of people or professionals in designing and implementing interventions.^(14)^ It has benefits such as improving creativity and idea generation, improving cooperation, loyalty to organizations and care processes, and better satisfaction and outcomes.^(13)^ These benefits are observed because co-design goes beyond just a participatory approach to empower stakeholders to the level of collaborators and decision-makers, thus improving ownership of a process, research, or an intervention.^(15)^

### Study timeline

There will be a three-month period of pre-implementation data collection to establish a baseline assessment of processes of care, population characteristics, and clinical outcomes. Active implementation will occur over a 6-week period, during which the research team will meet weekly with clinical stakeholders to identify potential barriers to implementation and develop solutions, including protocol adaptations and implementation strategies. After this time, the research team will step back from weekly meetings, with only data collection by trained data collectors, so as to evaluate the study outcomes.

### Data collection

The study requires data collected across the patient continuum, from the emergency unit through acute care to wards and the intensive care unit (ICU). Given the need for process (timeseries) data alongside one-off information such as patient characteristics and clinical outcomes, the potential burden of data collection is high. To help mitigate this, existing data sources will be used where possible. In the emergency unit, a recently established HER and triage scoring system will be leveraged to identify data on presentation, case mix, cohort identification, and risk assessment. Similarly, an existing digital ICU registry established in both adult and neonatal ICUs in the year 2022 will provide data on case mix, care processes, and risk-adjusted outcomes for the study cohort. Study-specific measures for implementation and care processes will be collected using a predetermined electronic Case Report Form (e-CRF) built in REDCap.^(16)^ The e-CRF will be developed in collaboration with stakeholders and piloted for feasibility testing prior to use.

### The study team

A purposively recruited stakeholder team will be convened to guide co-design, adaptation, and ratification of the clinical protocol (Table 1S - Supplementary Material). The team will include representation from trained specialist Obstetricians, Nurses/Midwives, Anesthesiologists, critical care intensivists, and a patient/patient relative. The research and implementation team is led by a trained intensivist working in the setting, with a special interest in maternal and obstetric emergency and critical care. He oversees a team of trained research assistants and a study coordinator, who will lead implementation and data collection. Methodological support for the design and evaluation of the study is provided by the lead intensivist's PhD supervisory team, who bring expertise in implementation science, stakeholder consultation methods, critical care, and statistical analysis.

### Protocol selection and adaptation

**Figure 1:**
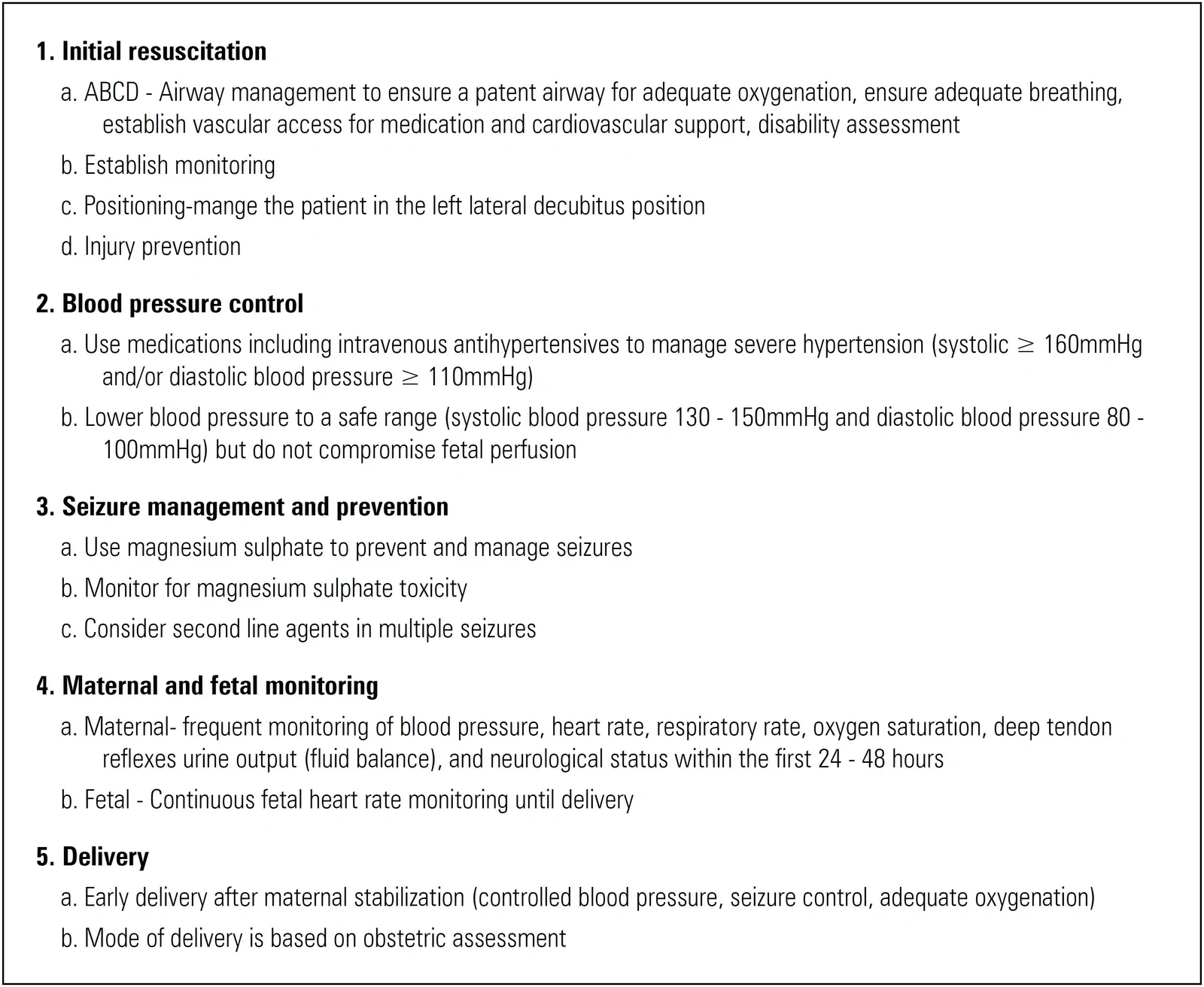
Key features of the protocol for managing eclampsia and pre-eclampsia with severe features.

The process will involve three stages: protocol selection and adaptation, implementation of the clinical protocol, and evaluation of the process (Figure 1S - Supplementary Material). First, the stakeholder team will draft, discuss, and critically review a setting-adapted protocol for managing pre-eclampsia with severe features and eclampsia. The drafting of the protocol will be guided by the process prescribed by the ADAPTE Collaboration Network titled Guideline Adaptation: A Resource Toolkit^(17)^ as used by Salarvand et al.,^(18)^ which involves three phases: set up, adaptation, and feedback. In the setup phase, the team will define the scope of work to be done. This will be followed by the Adaptation phase, which will involve an extensive systematic search of the literature to identify published guidelines (World Health Organization [WHO], Cochrane reviews, systematic reviews, randomized controlled trials (RCTs), and guidelines from reputable societies) for adoption or adaptation. The guidelines will then be adapted/revised to reflect current evidence and address the barriers to implementation and use identified during the scope. In the final phase, feedback will be obtained from wider stakeholders responsible for the management of patients with severe pre-eclampsia and eclampsia to ensure likely acceptability and identify potential barriers to implementation. Wider engagement at this stage of the design will be critical in engaging the clinical team, generating tension for change, and identifying and garnering buy-in from potential early adopters.^(19)^

**Figure 2:**
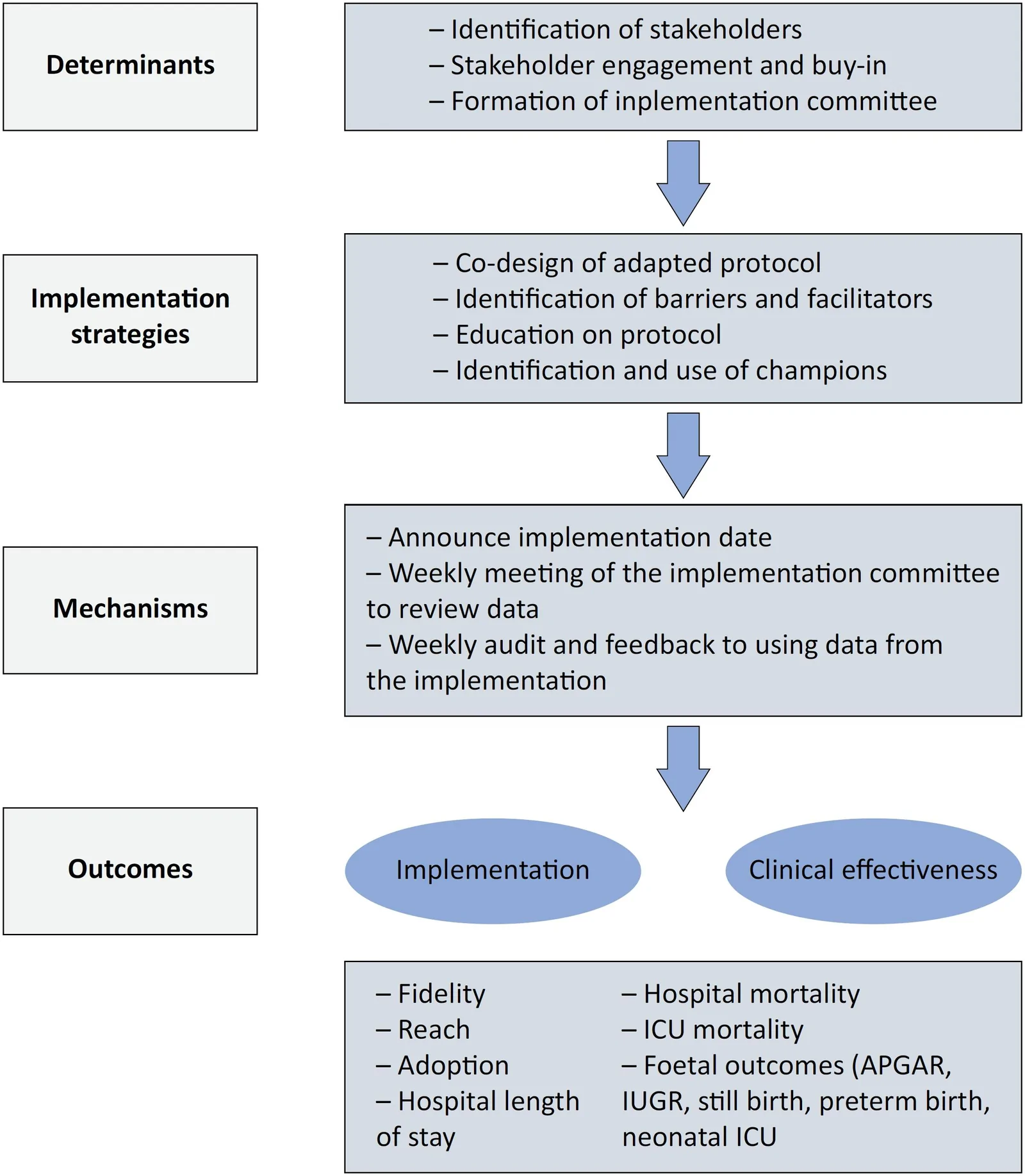
Logic model for implementation.

After drafting the protocol, a list of key processes will be identified as critical to the likelihood of successful implementation. These processes will be used to refine and improve a logic model, within which implementation outcomes will be measured (Figure 1). The logic framework will be guided by the Implementation Research Logic Model (IRLM), as depicted in figure 2.

### Implementation of the clinical protocol/guidelines

Once developed, the clinical protocol for the management of pre-eclampsia with severe features and eclampsia will be implemented in the clinical setting.

To improve the chances of implementation success, a combination of implementation strategies has been selected, given their superior effect in combination, when compared to the use of a single strategy.^(20)^ Strategies will include education, audit and feedback, and the use of local champions to guide implementation, adaptation, and tailoring. These strategies have been demonstrated to be feasible and effective in Lower- and Middle-Income Countries, but have not been evaluated in the context of emergency obstetric and maternal care in Ghana.^(21)^

A stakeholder is defined as any group or individual who is responsible for or affected by health- and healthcare-related decisions that can be informed by research evidence.^(22)^ In this study, the key stakeholders will be management members and clinicians (Obstetricians, Nurses/midwives, and Pharmacists). Extensive stakeholder engagement before an intervention can lead to a better understanding of barriers and local needs, ultimately improving the chances of successful implementation and adoption.^(23)^ The stakeholders will be informed about and educated on the project, its objectives, and the implementation plan.

### Education

Education in implementation science ensures that key stakeholders have adequate knowledge and information about an intervention, thereby improving uptake.^(24)^ The newly adapted clinical protocol for the management of pre-eclampsia with severe features and eclampsia will be shared with all clinical professionals (Nurses/Midwives, Doctors, and Pharmacists) in the Directorate of Obstetrics and Gynecology of the Komfo Anokye Teaching Hospital to study. Subsequently, training will be organized for all clinical professionals directly involved in the care of this patient cohort, in groups of 20 - 30. During the training, participants will be taken through the protocol's objectives and benefits, their roles, and the implementation process. The training will be completed a week before the start of the protocol implementation.

### Audit and feedback

Audit and feedback is an implementation strategy known to affect clinicians^’^ behavior^(25)^ and is widely applied in clinical care.^(26-28)^ A Cochrane review determined that it is an effective implementation strategy that enhances the adoption of interventions.^(29)^ During the implementation period, clinicians and stakeholders will receive selected clinical data periodically (every 2 weeks) to assess the progress of the implementation. The data will be presented in a report, developed from the study and registry data, and made available directly to the clinical team. The report will combine existing monthly data on case mix and clinical outcomes with study-specific data on implementation and care processes identified below.

### Implementation champion

Champions are individuals or healthcare providers who volunteer to promote the uptake or implementation of an intervention.^(30,31)^ Champions will be identified and utilized because their involvement has been found to facilitate the implementation of interventions.^(32)^ A nurse or midwife and a doctor will be identified as champions to facilitate the implementation. They will be tasked with encouraging their colleagues to adhere to the protocol and continuously explaining the benefits to them. They will also take note of any challenges that are encountered by their colleagues and report them to the implementation committee.

### Evaluation of implementation success/failure and impacts on clinical outcomes

The RE-AIM framework will be used to evaluate the implementation of the protocol intervention. RE-AIM framework offers a systematic evaluation of the effectiveness and impact of healthcare interventions. It is increasingly used both in Hybrid trials and to support implementation evaluations alongside more classical RCTs.^(12)^ In addition, interviews with stakeholders will be conducted prior to and following implementation. The interviews will explore stakeholder perceptions of protocolized care and identify behavioral factors that may influence implementation success. Interviews will be conducted by the research team, online, and de-identified, and transcribed prior to analysis.

### Primary outcome

The primary outcome will be implementation success, which will be assessed using three components of the RE-AIM framework (Fidelity, Reach, and Adoption). For each component of the framework, a threshold of 80% compliance across all three components will be considered success. This threshold is based on similar thresholds used in other studies and the understanding that, for policy implementation to have a meaningful impact on clinical outcomes and be sustainable as a long-term practice change, its adoption should be achieved in the greatest number of eligible patients as possible.^(33)^

### Secondary outcome

The secondary outcome will be the the intervention's effect on clinical care processes and outcomes associated with the care of patients with pre-eclampsia and eclampsia. These are based on a priori understanding of existing policies and on published eclampsia research,^(34)^ such as the PHOENIX trial.^(35)^ Selected clinical care processes and outcomes are: blood pressure monitoring, request of relevant laboratory investigations, time to administration of antihypertensives, time to intravenous administration, antihypertensive administration (in minutes), magnesium sulfate, volume of fluid administration, and the use of intravenous antihypertensives. In addition, outcomes for both the fetus^(35)^ (neonatal ICU admission, preterm birth, Apgar score, placental abruption, intrauterine fetal death (IUFD), intrauterine growth restriction (IUGR), mortality) and the mother^(34)^ (ICU admission, length of hospital stay, mortality, and neurological deficit) will also be assessed.

### Analysis plan

#### Patient demographics and patient characteristics

Descriptive statistics (mean/median) will be used to describe baseline patient characteristics: age, gravidity, parity, and blood pressure. The patient categories of antepartum and postpartum will be reported as percentages. The presenting diagnoses of the patients will also be reported as percentages.

#### Primary outcome

The individual components of Reach, Fidelity, and Adoption of the RE-AIM framework will be computed as percentages, as shown in table 1. To assess fidelity, a checklist of key clinical interventions will be developed from the protocol and compared to the care delivered. This will include the frequency of blood pressure monitoring, the threshold for administering intravenous antihypertensives, and the timeliness of magnesium sulfate administration. Fidelity, reach, and adoption will be equally weighted, so a composite computation will be done using the mean. A threshold of 80% in each of the three domains will define success.

**Table 1:** Primary and secondary outcome variables

Outcome	Measure and reporting
Implementation (primary outcome)	Fidelity: proportion of patients who are correctly managed with the protocol Numerator: number of patients who are correctly managed with the protocol Denominator: number of patients diagnosed with pre-eclampsia with severe features and eclampsia
	Reach: the proportion of patients who get managed with the protocol Numerator: number of patients who get managed with the protocol Denominator: number of obstetric emergency patients who presented at the Emergency Unit with the diagnosis of pre-eclampsia with severe features and eclampsia
	Adoption: proportion of patients who get managed with the protocol 6 months after the implementation Numerator: number of patients who get who get managed with the protocol in the 6th month of implementation Denominator: number of eligible patients who present to the Emergency Unit
Intervention effect and patient safety (secondary outcomes)	**Maternal** Hospital length of stay (in days) ICU referral and admission (expressed as a %) Hospital mortality (expressed as a %) ICU mortality (expressed as a %) Time to antihypertensive administration (in minutes; defined as the time between admission at the Emergency Department and the time of first administration of antihypertensive) Time to intravenous antihypertensive administration (in minutes; defined as the time between the first recorded blood pressure ≥ 180/110mmHg and the time of first administration of intravenous antihypertensive) Daily volume of fluid administration (expressed in mL/day) Time to magnesium sulphate administration (in minutes; defined as the time between admission at the Emergency Department and the time of first administration of magnesium sulphate)
	**Fetal** Neonatal ICU admission (expressed as a %) [Numerator = number of babies admitted to neonatal ICU, denominator = number of babies delivered] Preterm birth (expressed as a %) [Numerator = number of preterm deliveries, denominator = number of babies delivered] Placental abruption (expressed as a %) [Numerator = number of placental abruption, denominator = number of babies delivered] IUFD (expressed as a %) [Numerator = number of preterm intrauterine fetal deaths, denominator = number of babies delivered] IUGR (expressed as a %) [Numerator = number of IUGR, denominator = number of babies delivered] Average Apgar score at 1 and 5 minutes

ICU - intensive care unit; IUFD - intrauterine fetal death; IUGR - intrauterine growth restriction.

#### Qualitative analysis

Perceptions of individual and behavioral factors influencing the implementation of the protocol will be analyzed thematically from the interview transcriptions. Ten clinicians (obstetricians, anaesthesiologists, intensivists, midwives, critical care nurses) who are involved in the care of this cohort of patients will be interviewed before and after the implementation of the protocol. Barriers and facilitators will be analyzed using the Consolidated Framework for Implementation Research (CFIR 2).^(36)^ The CFIR 2 is ideally suited to facilitating identification, coding, and synthesis of context-specific behaviors, perceptions, and beliefs that may influence implementation.

To ensure reliability, transcripts will be analyzed by two researchers independently under the direct supervision of two researchers, who have extensive experience as researchers. The transcripts will first be reviewed line by line to generate open codes. The codes will then be analyzed to create categories of related codes. Further analysis of the categories will yield themes mapped to the CFIR 2 framework. These themes would then be used as part of a complementary analysis in understanding the quantitative RE-AIM findings.

#### Secondary outcomes

The secondary outcome variables will be measured in the pre-implementation, peri-implementation, and post-implementation periods. The details of the outcome variables have been listed in table 1. Changes in measure (i.e., time, therapy, event rate) will be reported descriptively. The study is not powered to detect a significant change associated with the intervention; rather, these clinical process and outcome measures will provide a signal for safety and provide important feasibility information to inform future scalable studies if the protocol and co-design approach to implementation is found to be effective.

## DISCUSSION

Pre-eclampsia and eclampsia are among the top five causes of maternal mortality in Sub-Saharan Africa.^(37)^ In Ghana, it is one of the top two causes of maternal mortality.^(38)^ Given its significant contribution to maternal mortality, any intervention that improves maternal outcomes in that cohort of patients will considerably impact overall maternal outcomes.

Standardized policies and protocols for clinical care have been shown to improve outcomes,^(39)^ but the generally poor uptake requires the use of systematic processes to overcome implementation barriers and identify potential facilitators.

Studies have identified barriers to the adoption of evidence-based practices in maternal and obstetric care. These include: staffing gaps, lack of clinical knowledge, lack of resources, lack of organizational support, and behaviors and beliefs that may lead to guidelines being perceived as competing with other clinical priorities or in conflict with clinician autonomy.^(1,2)^ Furthermore, a lack of buy-in from medical staff and inadequate stakeholder needs assessment may also limit implementation and adoption.^(40)^

This study is designed to assess the effectiveness of implementation strategies and stakeholder co-design in improving the management of pre-eclampsia and eclampsia. Improving management and outcomes for this population requires healthcare teams from different specialties to work collaboratively, often rapidly (i.e., within the first hour of admission), and to support ongoing management, often during the transfer of patients between emergency units, operating theatres, and, sometimes, intensive care.^(41)^ The complexity of this patient care pathway means it is critical to identify, and hopefully mitigate potential barriers to policy adoption and practice change, prior to wider scaled implementation. Furthermore, this patient population is vulnerable, and both mother and baby are exposed to significant risk of morbidity and mortality if the condition is not optimally managed.^(42)^

This study will generate new information regarding the effect of these strategies in the emergency, obstetrics, and maternal care population, drawing on perceptions and experiences from a diverse healthcare team. carefully designed with active stakeholder participation to overcome known barriers in implementation science. Stakeholder engagement before implementing a protocol enable the identification of context-specific barriers and givefosters clinicians’ sense of ownership, which improves the chances of success.^(23)^ Some Doctors and Nurses will be identified as champions to leverage their influence in the department to enhance implementation as documented in literatutre.^(31)^ Throughout the implementation period, progress will be audited periodically through data analysis to provide feedback to clinicians, as audit and feedback positively influence clinicians’ attitudes towards the implementation.^(2)^ The strategies to enhance implementation (education, stakeholder engagements, use of champions, audit, and feedback) will be employed over six weeks of active implementation. Their eventual withdrawal may pose a challenge to sustainability, but it is expected that the potential benefits in clinical outcomes and systems improvement would drive sustainability.

The success of these implementation strategies will be evaluated using the RE-AIM framework's 3 domains (fidelity, reach, and adoption). The RE-AIM framework is an effective tool for systematically evaluating healthcare interventions.^(32)^

Despite this study's purposeful selection of strategies to support context adaptation and engagement from clinical stakeholders, there is no assurance of success. The findings of the study will determine the ease of adoption and implementation of this clinical protocol, identify barriers and facilitators of the implementation process, and assess the impact on maternal outcomes specific to both the hospital and potentially relevant to the wider Ghanaian healthcare system. Though a single-center study, the findings may still serve as a guide for future policy implementation on a broader scale.

## Data Availability

The contents underlying the research text are included in the manuscript.
