# Supplementary material for: Adaptation and implementation of a clinical protocol for pre-eclampsia with severe features and eclampsia in a teaching hospital in Ghana: a study protocol
Source: Crit Care Sci. 2026 Jul 21;38:e20260312. doi: 10.62675/2965-2774.20260312 (PMC13518025; doi:10.62675/2965-2774.20260312)
Supplement: Supplementary file 1 [file 2965-2774-ccsci-38-e20260312-Suppl01.pdf]

Critical Care Science
https://doi.org/10.62675/2965-2774.20260312

SUPPLEMENTARY MATERIAL

Adaptation and implementation of a clinical protocol for pre-eclampsia with severe features and eclampsia in a teaching hospital in Ghana: a study protocol

Moses Siaw-Frimpong[1], Abigail Beane[2], Rashan Haniffa[2], Jorge Ibrain Figueira Salluh[3], John Humphrey Amuasi[4], Yaw Gyanteh Owusu[5], Fathima Fazla[6], Joseph Bonney[7], Ibrahim Kwaku Duah[7], Patience Adjepong[1], Nana Fosua Gyapon[1], Irene Bandoh[1], William Addison[1], Andrew Panyin Vormawor[5], Charles Adu-Takyi[5], Augustine Tawia[5]

Table 1S - Scope of work and the meeting plan of the implementation committee

Meeting number	Proposed agenda	Key outputs
1	Overview of the task/scope of work (members will be briefed on the entire project, stating the aims and the objectives. The specific task of the committee, which is the selection and adoption of the protocol, identifying barriers and facilitators for the implementation, and implementing the protocol, will be explained to the members):<br>– Identifying and sharing the list of published protocols to be adopted<br>– It will be explained that reputable organizations and medical societies must publish the protocol to be adopted. These will include the WHO,(1) NICE,(2) FIGO, SOGOG etc.)<br>– These resources will be shared at the end of the meeting for review<br>– The factors that will guide the review will include; resource availability, feasibility of implementation, and skills mix required to implement the protocol, mainly determined by the site/ward where the patient is admitted - location and monitoring, as well as patient safety endpoints<br>– Generally, the protocol must be simple and not lead to a significant increase in the stress of work	Members will be tasked to review the protocols before the next meeting
2	Report on the reviewed protocol on the following:<br>– Summary of the population<br>– Inclusion and exclusion criteria<br>– Therapy-routes and doses<br>– Monitoring<br>– Patient safety endpoints<br>The finalized draft of the adapted protocol will be sent to the leadership of the department for review by senior doctors and midwives	Adapted local protocol
3	– Incorporating the feedback from the department review by senior doctors and midwives<br>– Review of the finalized protocol<br>– Planning dissemination and feedback<br>– Finalizing a list of key stakeholders from whose feedback will be obtained to identify potential barriers and facilitators<br>– Sharing tasks on stakeholder engagements<br>The protocol will be reviewed by stakeholders to identify a list of facilitators and barriers. The stakeholders will include all the members of the committee plus 30 additional people (10 obstetricians, 10 nurses/midwives, 5 anaesthesiologists, 2 intensivists, 2 pharmacists, and the Head of the Obstetric Department)<br>During the meeting, views on every item in the protocol will be taken to list potential barriers and facilitators<br>Some key questions will be asked to assess the categories of barriers and facilitation guided by Wändell et al.:(3)<br>– Structural (time restraint of the protocol implementation, sufficient support for the implementation)<br>– Organizational (clearly defined roles in patient care, structure of supervision, Information Technology support, logistics support)<br>– Professional (adequate knowledge and experience in pre-eclampsia and eclampsia among staff, adequate training)<br>– Patient-related (perception of patient harm, good doctor-patient relationship to increase patient acceptability)<br>– Attitudinal (desire of clinicians to improve outcomes and prevent harm, attitudes towards change)<br>When disagreements exist, the methods that would be employed to resolve them will include:<br>– Taking a majority decision through voting<br>– Taking more views from other professionals who are not part of the stakeholders	Finalized protocol
4	The implementation committee at the last meeting will discuss and finalize the following:<br>– Consolidating the list of barriers and identification<br>– Identifying champions<br>– Drafting a schedule for the education of staff on the new protocol<br>Decide the implementation date	A list of barriers and facilitators<br>Identification of champions<br>Implementation strategy

WHO - World Health Organization; NICE - National Institute for Health and Care Excellence; FIGO - International Federation of Gynecology and Obstetrics; SOGOG - Society of Obstetricians and Gynecologists in Ghana.

This is an open access article under the CC BY license (https://creativecommons.org/licenses/by/4.0/).

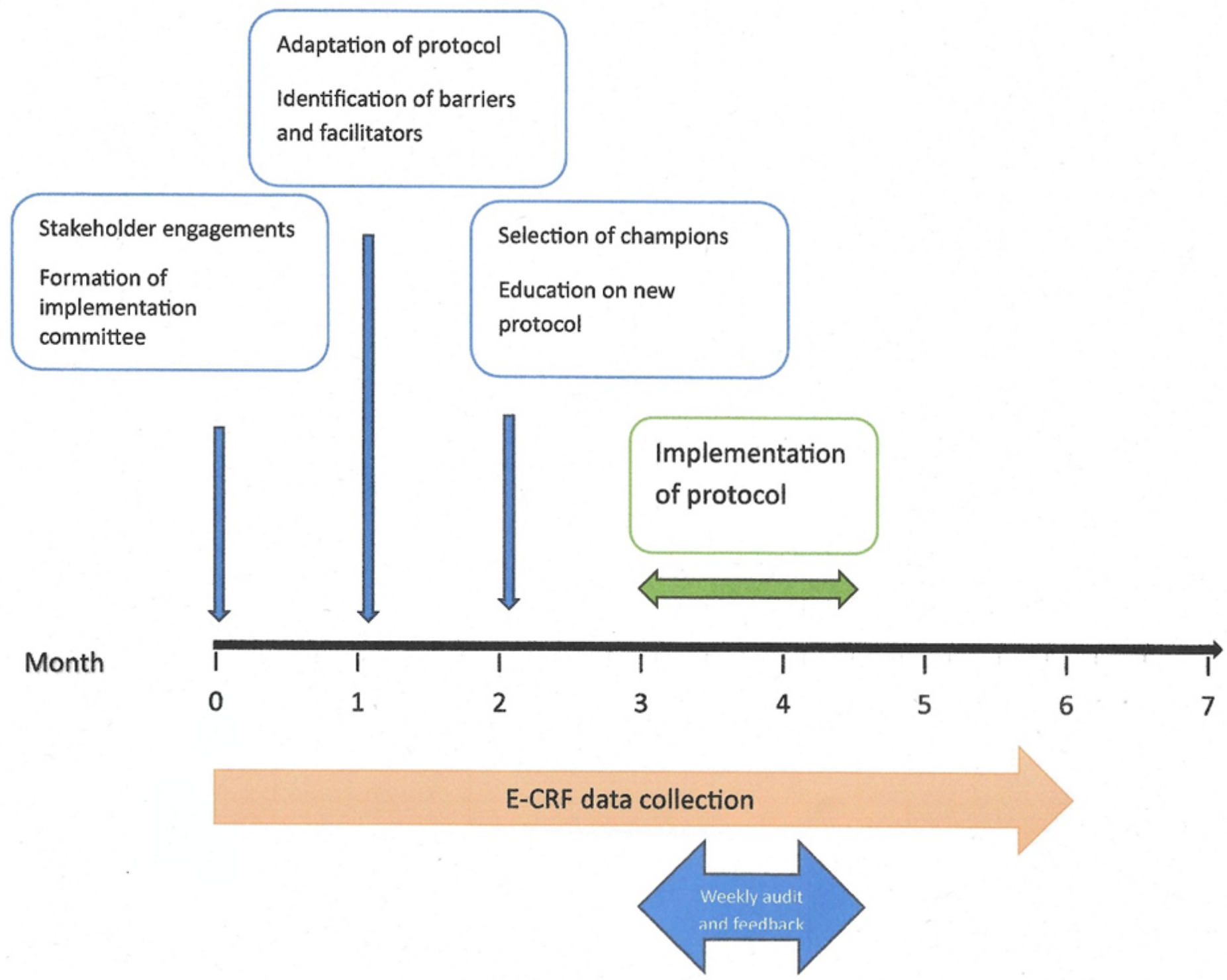

E-CRF - Case Report Form.

Figure 1S - Schematic diagram of the implementation process.

REFERENCES

1. World Health Organization (WHO). WHO recommendations: drug treatment for severe hypertension in pregnancy. Geneva: World Health Organization; 2018. Available from: https://iris.who.int/server/api/core/bitstreams/cee6df5c-d3b4-4097-af7a-405428dd221c/content
2. National Institute for Health and Care Excellence (NICE). Hypertension in pregnancy: diagnosis and management. NICE guideline. Available from: https://www.nice.org.uk/guidance/ng133/resources/hypertension-in-pregnancy-diagnosis-and-management-pdf-66141717671365
3. Wändell PE, de Waard AM, Holzmann MJ, Gornitzki C, Lionis C, de Wit N, et al. Barriers and facilitators among health professionals in primary care to prevention of cardiometabolic diseases: a systematic review. Fam Pract. 2018;35(4):383-98.
